# Strapdown Inertial Navigation Systems for Positioning Mobile Robots—MEMS Gyroscopes Random Errors Analysis Using Allan Variance Method

**DOI:** 10.3390/s20174841

**Published:** 2020-08-27

**Authors:** Andrii V. Rudyk, Andriy O. Semenov, Natalia Kryvinska, Olena O. Semenova, Volodymyr P. Kvasnikov, Andrii P. Safonyk

**Affiliations:** 1 Department of Automation, Electrical Engineering and Computer-Integrated Technologies, National University of Water and Environmental Engineering, Soborna Street 11, 33000 Rivne, Ukraine; a.v.rudyk@nuwm.edu.ua (A.V.R.); a.p.safonyk@nuwm.edu.ua (A.P.S.); 2 Faculty for Infocommunications, Radioelectronics and Nanosystems, Vinnytsia National Technical University, Khmelnytske shose 95, 21000 Vinnytsia, Ukraine; semenov.a.o@vntu.edu.ua (A.O.S.); semenova.o.o@vntu.edu.ua (O.O.S.); 3 Department of Industrial Engineering, University of Applied Sciences - Technikum Wien, 1200 Vienna, Austria; 4 Department of Information Systems, Faculty of Management, Comenius University in Bratislava, 82005 25 Bratislava, Slovakia; 5 Department of Computerized Electrotechnical Systems and Technologies, National Aviation University, Prospect Kosmonavta Komarova, 1, 02000 Kyiv, Ukraine; kvp@nau.edu.ua

**Keywords:** mobile robots, strapdown inertial navigation systems (SINS), gyroscope, error equation, Allan deviation, bias instability, angle random walk

## Abstract

A problem of estimating the movement and orientation of a mobile robot is examined in this paper. The strapdown inertial navigation systems are often engaged to solve this common obstacle. The most important and critically sensitive component of such positioning approximation system is a gyroscope. Thus, we analyze here the random error components of the gyroscope, such as bias instability and random rate walk, as well as those that cause the presence of white and exponentially correlated (Markov) noise and perform an optimization of these parameters. The MEMS gyroscopes of InvenSense MPU-6050 type for each axis of the gyroscope with a sampling frequency of 70 Hz are investigated, as a result, Allan variance graphs and the values of bias instability coefficient and angle random walk for each axis are determined. It was found that in the output signals of the gyroscopes there is no Markov noise and random rate walk, and the X and Z axes are noisier than the Y axis. In the process of inertial measurement unit (IMU) calibration, the correction coefficients are calculated, which allow partial compensating the influence of destabilizing factors and determining the perpendicularity inaccuracy for sensitivity axes, and the conversion coefficients for each axis, which transform the sensor source codes into the measure unit and bias for each axis. The output signals of the calibrated gyroscope are noisy and offset from zero to all axes, so processing accelerometer and gyroscope data by the alpha-beta filter or Kalman filter is required to reduce noise influence.

## 1. Introduction

Strapdown inertial navigation systems (SINS) are often employed to solve a problem of estimating movement and orientation of a mobile robot (MR). These systems include accelerometers, gyroscopes, and magnetometers, if needed. Gyroscopes are utilized to determine the MR orientation angles, which are relative to an inertial frame of reference. Accelerometers measure the object acceleration along the axes of the Cartesian coordinate system. Therefore, the MR rotation relative to the vertical axis is determined applying the gyroscope, the speed and distance traveled are determined by the results of integrating the measured values of acceleration [1,2].

Installed in the MR board gyroscopes and accelerometers must have an adjusted accuracy in a wide range of angular velocities and accelerations and operate under conditions of vibrations and hits. The number of sensors should be sufficient to obtain information about the imaginary acceleration vectors and the absolute angular velocity of the MR.

Application of a gyroscope system provides all three angles (roll, pitch, and yaw). For a terrestrial MR, the most interesting is the angle of rotation about a vertical axis, is the yaw. However, a practical implementation faces some nuances of using gyroscopes in MRs [3,4].

Firstly, it is impractical to install mechanical gyroscopes in MRs because of their large size. Therefore, miniature gyroscopes in a form of microelectromechanical systems (MEMS) are employed, their informative parameter being an angular velocity. 

In this case, integration or simple summation (for analog or discrete output) are performed. Therefore, the estimate of rotation about an axis is approximate and depends on a signal sampling frequency [5,6]:

(1) $$\alpha \left(t\right)=\int_{0}^{t}\omega \left(t\right)dt,\;\alpha_{i+1}=\alpha_{i}+\omega_{i}\Delta t.$$

Secondly, gyroscopes are characterized by zero drift, leading to changes in angle even in a static position. The drift magnitude depends on a type of the gyroscope [7]. Error ranges for different types of sensors are given in [8].

Thirdly, integration and processing of inertial sensor data with a frequency required for the sufficient accuracy creates a high computational load that might need a separate microcontroller.

Accelerometers in the inertial system provide determining values of linear accelerations for the MR. Numerical integration of acceleration allows passing to speed, and repeated integration allows passing to movement during any time interval [9]. However, integration leads to the accumulation of error [10]. Moreover, accelerometers are sensitive to intense high-frequency interferences, which eliminated by various types of filters (the Kalman filter, alpha-beta filter, etc.). However, such filters are difficult to implement, they require selection or calculation of coefficients and need considerable resources to be implemented on microcontrollers.

While analyzing the SINS accuracy characteristics caused by the gyroscope errors, attention is paid to zero instability (offset) and residual systematic errors. This paper focuses on the instability of gyroscope’s zeros, which typically represent a sum of systematic $\delta \omega_{S}$ (Systematic Error) and random $\delta \omega_{R}$ (Random Error) components [11]:

(2) $$\delta \omega =\delta \omega_{S}+\delta \omega_{R}.$$

The cause of the input signal shift in the gyroscope is parasitic moments effecting moving parts of the sensor and electronic components unbalance in systems of information reading and processing.

Problem statement. The article is aimed to:

1. Analyze the components of the gyroscope random error due to the influence of various destabilizing factors.
2. Study the MEMS gyroscopes in order to determine a value of their random error components for each axis of Allan deviation curves.

## 2. Methods of Analyzing Random Errors in Gyroscopes 

Frequency analysis methods considering power spectral density (PSD) [11] and temporal analysis techniques considering the Allan variance (AVAR) [12,13,14,15] are applied to examine the gyroscope random errors.

The power spectral density $S\left(\omega \right)$ is defined as the bilateral Fourier transform of a correlation function $K\left(\tau \right)$. The Allan variance is a method of time sequences analysis for determining noise characteristics as a function of average time and can be found by results of recording and output signal $\tilde{u}\left(t\right)$ from the gyroscope, based on a stationary platform. The gyroscope’s output signal recording consists of *N* samples with duration $T_{0}$. The total length the recording is $NT_{0}$. This method was originally developed to evaluate standard frequency errors. Later, it was actively used for estimating parameters of inertial sensitive elements (recommendations to use this method of analysis are given in the IEEE standards [12,13].

According to this method, an initial array of *N* measurements of an angular velocity $\tilde{\omega }\left(t\right)$ is received after the test measurements. Then the array is divided into *K = N/M* groups, where *M* is a size of the sequential measurements group. The angle accumulated by integrating results of a gyroscope angular velocity $\tilde{\omega }\left(t\right)$ at an interval of *M* samples is determined for each group.

(3) $$\theta_{k}=\int_{0}^{MT_{0}}\tilde{\omega }\left(t\right)dt,\;k=1,\;\ldots \;,\;K.$$

To calculate the Allan variance, one has to determine a dispersion of differences of the accumulated angles $\theta_{k}$ for two neighboring groups having a time shift at correlation time $\tau =M/f_{D}=MT_{0}$, where $f_{D}$ is a sampling frequency of gyroscope output signals:

(4) $$\sigma_{A}^{2}\left(\tau \right)=\frac{1}{2\;\left(K-1\right)}\sum_{k=1}^{K-1}\;{\left[\theta_{k+1}-\theta_{k}\right]}^{2}.$$

**Figure 1:**
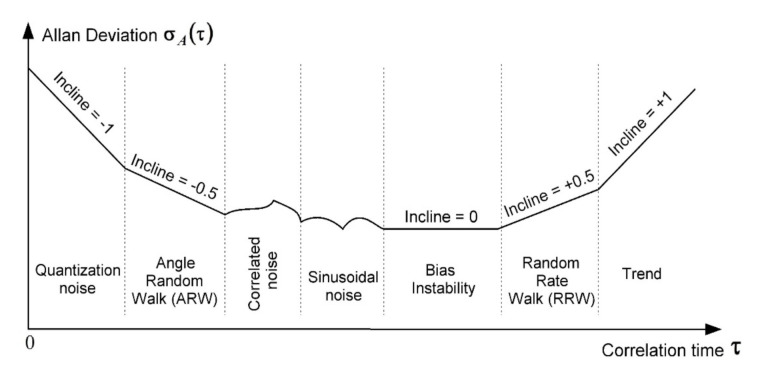
General view of the Allan deviation curve. The Allan deviation allow separating such noise components as quasi-deterministic zero offset (+1 inclination); gyroscope angular velocity random walk (+0.5 inclination); zero offset instability or flicker noise (0 inclination); random walk of gyroscope angle (−0.5 inclination); and output quantization noise (−1 inclination) [16].

However, in practice it is often not the Allan variance $\sigma_{A}^{2}\left(\tau \right)$ to be determined, but the Allan deviation (AD) $\sigma_{A}\left(\tau \right)$. Then, a curve of the Allan deviation against the average time is plotted. Different sections of the curve are analyzed. Finally, conclusions are drawn about the presence of various components of the gyroscope error (Figure 1) [16].

The gyroscope zero offset systematic error $\delta \omega_{S}$ from Equation (2) is the sum of the basic $\delta \omega_{SB}$ and additional $\delta \omega_{SA}$ systematic errors. Typically, the basic systematic error of a gyroscope will be different from the basic systematic error of another gyroscope of the same type. That is because a group of similar gyroscopes the main systematic error is considered as a random error, but constant in the operating cycle [11,17,18].

Additional errors of gyroscopes are related to their sensitivity to changes of external factors; they are:

- the sensitivity to acceleration $\delta \omega_{SA.a}a$, where $\delta \omega_{SA.a}$ is the coefficient of gyroscope sensitivity to acceleration against the respective axis ((rad∙s)/m) or ((rad/s)/g); a is the acceleration;
- the sensitivity to temperature changes $\delta \omega_{SA.t}\Delta t$, where $\delta \omega_{SA.t}$ is the coefficient of gyroscope sensitivity to temperature changes, (rad/(s∙°C); Δt is the temperature deviation from its norm;
- the sensitivity to vibration $\delta \omega_{SA.\nu }\nu$, where $\delta \omega_{SA.\nu }$ is the gyroscope sensitivity coefficient to a vibration frequency ((rad/s)/Hz) or (rad); ν is the vibration frequency;
- sensitivity variations that are not a function of the measured orientation angles, for example, those depending on climatic factors (temperature *T*, relative humidity *W,* and ambient air pressure *P*), which differ from their nominal values when measuring motion of objects *T*_0_ = 20 °C, *W_0_* = 65%, and *P*_0_ = 99.992 kPa (750 mm. mer. col.) (climate drift is $\Delta S_{\left(\Delta T,\;\Delta W,\;\Delta P\right)}$), depending only on temperature (temperature drift is $\Delta S_{\left(\Delta T\right)}$), or as a result of other factors during the time interval Δt (time drift is $\Delta S_{\left(\Delta t\right)}$):
  (5) $$\Delta S_{\left(\Delta T,\;\Delta W,\;\Delta P\right)}=\frac{S_{\left(T,\;W,\;P\right)}-S_{\left(T_{0},\;W_{0},\;P_{0}\right)}}{S_{\left(T_{0},\;W_{0},\;P_{0}\right)}}\cdot 100\%;$$
  (6) $$\Delta S_{\left(\Delta T\right)}=\frac{S_{\left(T\right)}-S_{\left(T_{0}\right)}}{S_{\left(T_{0}\right)}}\cdot 100\%;$$
  (7) $$\Delta S_{\left(\Delta t\right)}=\frac{S_{\left(t_{0}+\Delta t\right)}-S_{\left(t_{0}\right)}}{S_{\left(t_{0}\right)}}\cdot 100\%.$$

## 3. Analysis of the Gyroscope Random Error Components 

A generalized equation of gyroscope errors obtained in [11,19] is

(8) $$\Delta \omega =0.01\delta K^{G}\omega -\left[M^{G}\times \right]\;\omega +\delta \omega ,$$

where $\delta K^{G}={\left(K^{G}\right)}^{-1}\Delta K^{G}\cdot 100\%=diag\;\left\|\begin{array}{ccc} \;\delta k_{X}^{G} & \delta k_{Y}^{G} & \delta k_{Z}^{G} \end{array}\right\|$ is the diagonal matrix of relative errors of gyroscope conversion coefficients [%]; $\left[M^{G}\times \right]$ is the skew-symmetric matrix corresponding to the matrix of directional cosines $M^{G}$; $\delta \omega ={\left\|\begin{array}{ccc} \;\delta \omega_{X}^{u} & \delta \omega_{Y}^{u} & \delta \omega_{Z}^{u} \end{array}\right\|}^{T}$ is the gyroscope zero offset vector with dimension of this gyroscope output signal.

The first summand in ratio (8) describes the effect of conversion coefficient errors, the second one shows malfunctioning installation of gyroscope measuring axes, the third is the gyroscope zero offsets determined by ratio (2).

The random error components of gyroscopes, which are inertial sensors, are defined by the presence of noise and noisy processes of various kinds [11,20]:

(9) $$\delta \omega_{R}=\delta \omega_{WN}+\delta \omega_{BI}+\delta \omega_{RRW}+\delta \omega_{MN},$$

where $\delta \omega_{WN}$ is the random error component caused by white noise (*WN*); $\delta \omega_{BI}$ is the bias instability (*BI*); $\delta \omega_{RRW}$ is the random rate walk (*RRW*); $\delta \omega_{MN}$ is the of the random error component caused by the exponential correlated (Markov) noise (*MN*).

Various disturbances influence the gyroscope output signal. If the gyroscope signal is taken at discrete time points of the sampling period $T_{0}$, the gyroscope noise can be described as white sequence (discrete white noise) $\delta \omega_{WN}\left(k\right)$, k=1, 2, … with the zero average $M\left[\delta \omega_{WN}\left(k\right)\right]=0$ and the limited variance value $D\left[\delta \omega_{WN}\left(k\right)\right]=\sigma_{WN}^{2}$. The error variance determining the rotation angle is $D\left[\int_{0}^{t}\delta \omega_{WN}\left(\tau \right)d\tau \right]=T_{0}t\;\sigma_{WN}^{2}$ [11,21]. Here, the standard deviation (SD) of the error accumulation from the rotation angle determination was caused by the presence of white noise in the gyroscope output signal. Moreover, it increases proportional to the square root of the time:

(10) $$\sigma_{\theta }\left(t\right)=\sigma_{WN}\sqrt{T_{0}t}\;=\theta_{ARW}\sqrt{t}\;,$$

where $\theta_{ARW}=\sigma_{WN}\sqrt{T_{0}}$ is the angle random walk (*ARW*) with dimension $\dim\theta_{ARW}=\left[\mathrm{rad}/s^{1/2}\right]$ or dimθARW=o/sHz [22,23].

The criterion for the presence of white noise in the gyroscope output signal is the presence of a rectilinear section with inclination −0.5 on the Allan deviation curve (Figure 1); thus, the ARW is the Allan deviation at *τ* = 1 s. Moreover, accuracy of the random process parameter determination depends on the record length.

Bias instability (*BI*) is caused by noises in the electronic components of gyroscope systems of information reading and processing. It is relative to *Flicker noise* or 1/f noise having the following spectral density

$$S\left(\omega \right)=\left\{\begin{array}{l} \frac{B^{2}}{2\pi \omega },\;\omega \leq \omega_{0};\\ 0\;,\;\omega >\omega_{0}, \end{array}\right.$$

where $\omega_{0}$ is the cutoff frequency, and B is the coefficient of bias instability. The flicker noise is a universal type of fluctuations and exists almost in all materials and elements used in electronics. In the model of zero instability formation, the fractional integration of which the white noise spectrum is used, which is the half-order integral from the white noise $w\left(t\right)$. If white noise passes through a filter with the transfer function $K\left(p\right)=\frac{1}{p^{1/2}}$, fluctuations at the filter output will have the 1/f–spectrum. Such hypothetical filter acts as an integrator of fractional order [11].

**Figure 2:**
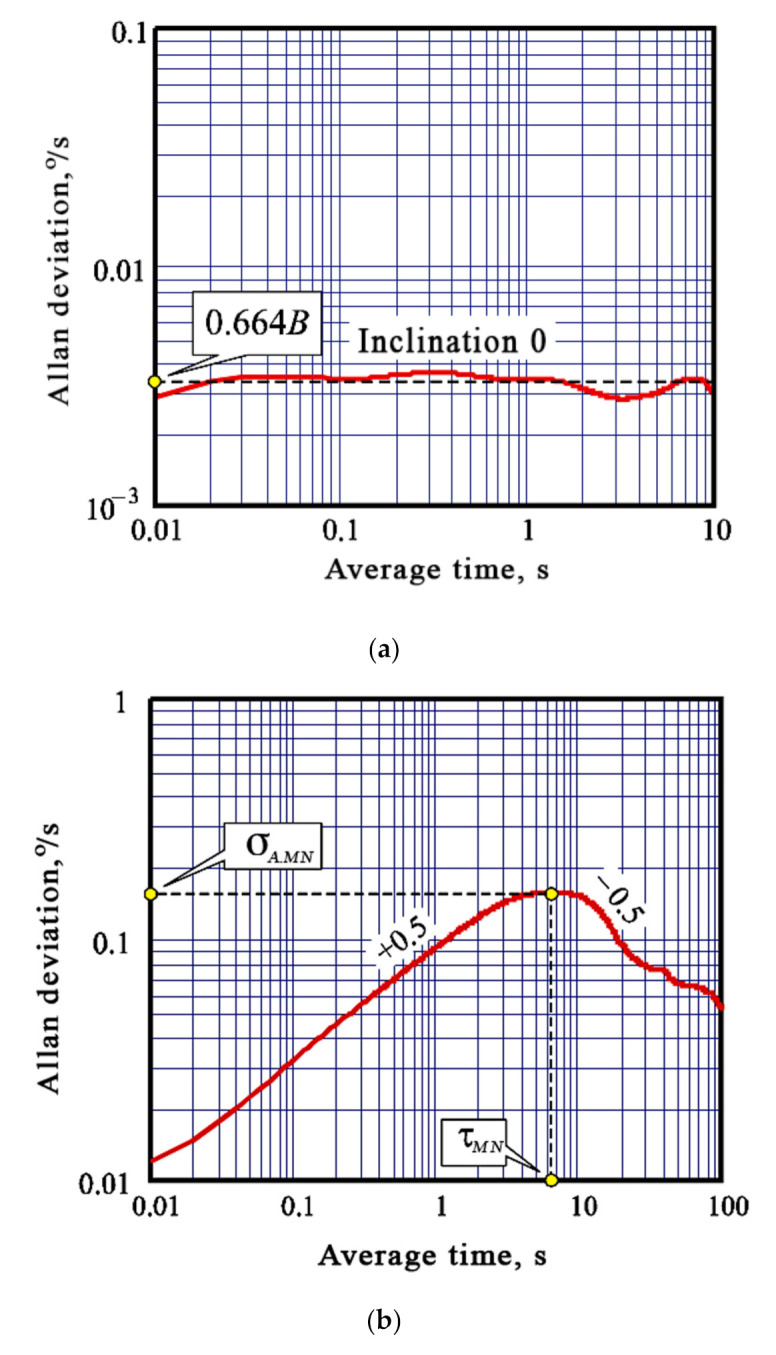
Determination of bias instability (**a**) and Markov noise parameters (**b**) by the Allan deviation curve.

The gyroscope BI coefficient [24] is determined by a section of the Allan deviation curve with inclination 0 (at the minimum value of the AD curve) (Figure 2a) [16,25]:

(11) $$B=\frac{\sigma \left(\tau =nT_{0}\right)}{0.664}$$

The random rate walk (RRW) $\delta \omega_{RRW}$ is described by the Wiener random process (random walk) and leads to the presence of a straight section with inclination +0.5 on the Allan deviation curve (Figure 1). In order to determine the spectral density of white noise $w\left(t\right)$ passed through the integrator to form the random walk, one has to find the AVAR at τ=3 s . The standard deviation of the RRW is accumulated over time similar to (8):

(12) $$\sigma_{RRW}\left(t\right)=\sigma_{WN}^{*}\sqrt{T_{0}t}\;=\omega_{RRW}\sqrt{t}\;,$$

where $\sigma_{WN}^{*}$ is the SD of a generating white noise $w\left(t\right)$.

In order to determine the correlation time $T_{MN}$ (or an attenuation coefficient of correlation function $\mu =T_{MN}^{-1}$ [s^−1^]) and the dispersion $D_{MN}$ (or SD $\sigma_{MN}$) of Markov noise the Allan variance method is applied. According to it, the Allan deviation curve (Figure 1) is used to determine the local maximum coordinates $\tau_{MN}$ and $\sigma_{A.MN}$ between asymptotes with inclinations +0.5 and −0.5 (Figure 2b) [16], and the required Markov noise parameters are specified as follows [11]:

(13) $$\sigma_{MN}=1.618\cdot \sigma_{A.MN},\;T_{MN}=0.529\cdot \tau_{MN}\;.$$

This method provides accuracy enhancement for the Markov noise parameters determination by increasing the noise recording length [26].

## 4. Experimental Examination of Noise Parameters of the InvenSense MPU-6050 Gyroscope 

Electrical characteristics (gyroscope and accelerometer specifications) of the InvenSense MPU-6050 are presented in Table 1 [27].

Experimental examination of noise characteristics for each axis of the MEMS gyroscope inertial measurement unit (IMU) type InvenSense MPU-6050 was performed with application of the Allan variation method for 1 h at temperature (22 ± 3) °C. Data from the gyroscope were taken at a frequency of 70 Hz and the module was stationary. The read data was written to a text file and imported into MatLab, where the Allan deviation was calculated.

**Figure 3:**
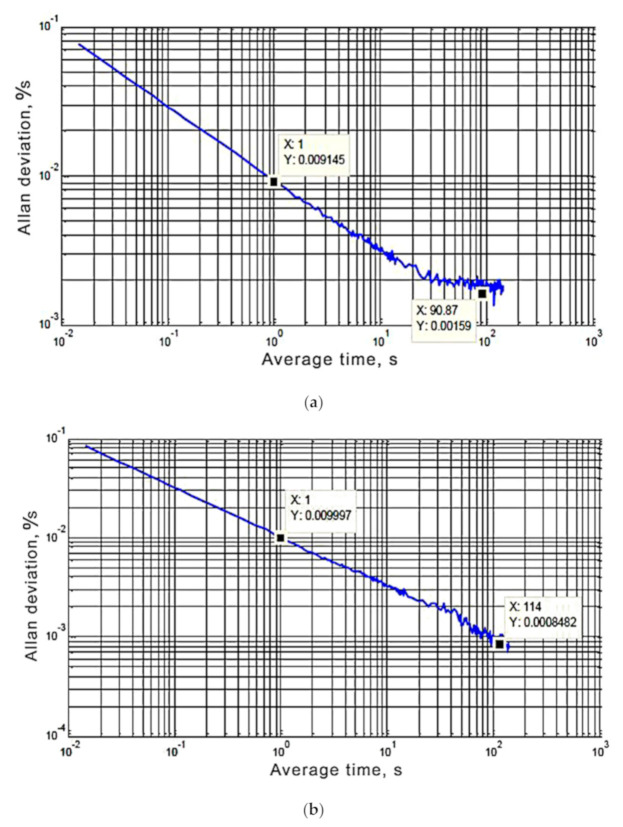

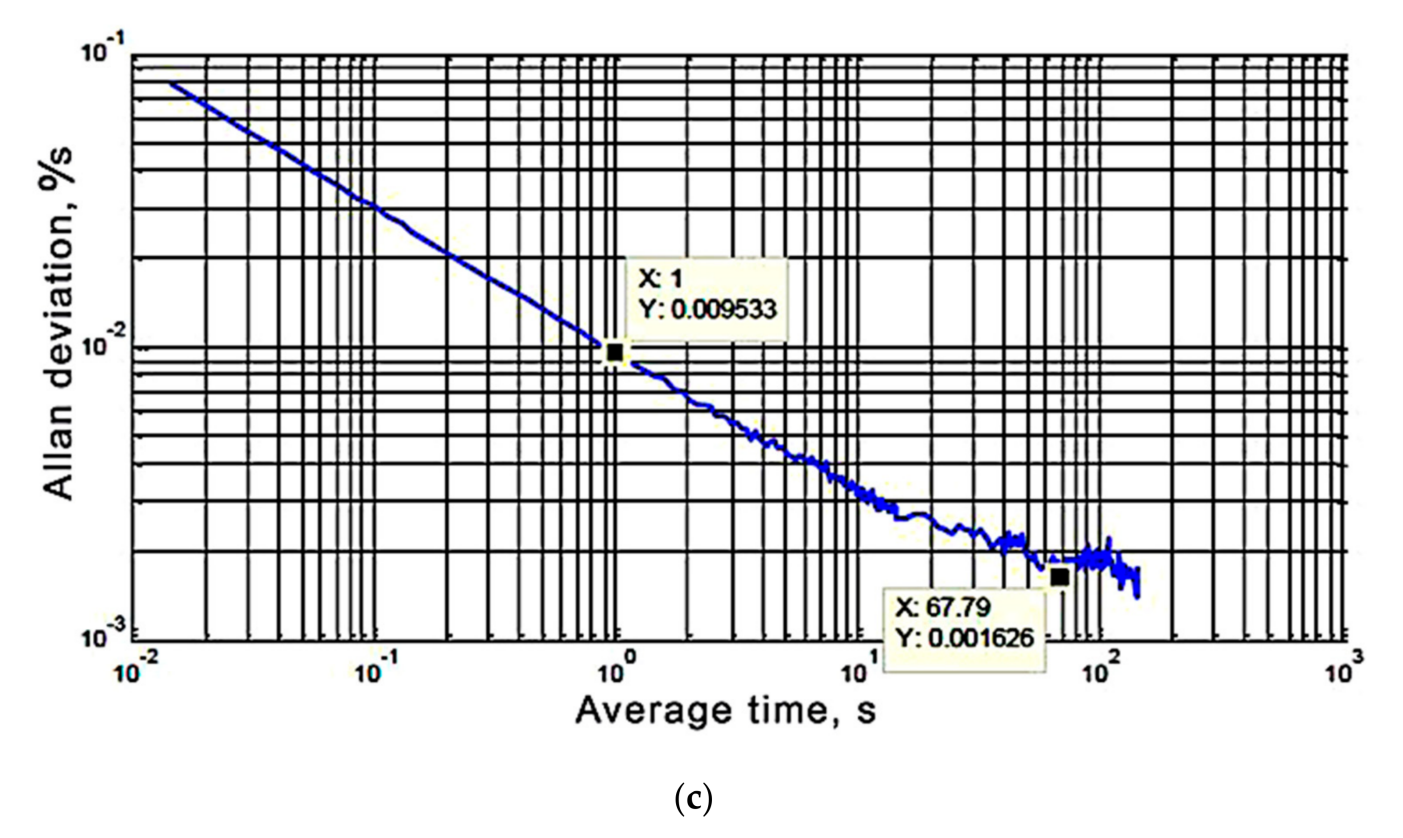
Graphs of the Allan deviation for the gyroscope sensor MPU-6050: axis X (**a**), axis Y (**b**), and axis Z (**c**).

The graphical dependences of the Allan deviation against the correlation time for gyroscope each axis are shown in Figure 3. As it was expected, at small values τ, there is an inclination −0.5 of the Allan deviation curves, this corresponds to white noise in the gyroscope output signals. The angle random walk $\theta_{ARW}$ is determined at τ=1 s. The Allan deviation curves have a minimum on the zero-slope section corresponding to the bias instability in the area $\tau =\left(20÷150\right)\;s$. Increase of the averaging time was not analyzed, so a positive inclination +0.5 was not detected, indicating that there is no random rate walk in the gyroscope outputs. A local maximum between the asymptotes with inclinations +0.5 and −0.5 is also not observed on the Allan deviation curves, this indicates the absence of Markov noise in the gyroscope output signals.

The angle random walk $\theta_{ARW}$ and the coefficient of bias instability *B* for each axis of the gyroscope IMU were determined by graphs of the Allan deviation against the correlation time (Figure 3).

θARW.X=0.009145 o/sHz ; θARW.Y=0.009997 o/sHz ; θARW.Z=0.009533 o/sHz ; σAG=0.664⋅B o/s ; BXG=0.001590.664=0.00239 o/s ; BYG=0.00084820.664=0.001277 o/s ; BZG=0.0016260.664=0.002449 o/s .

Analysis of results of the gyroscope noise parameters measurement shows that the X and Z axes have the twice bigger bias instability coefficient than the Y axis. According to preliminary conclusions, this was caused by measurement errors of gyroscope scale factors. However, in similar repeated experiments, it was found that the gyroscopes scale factors were correctly calculated, their axes are aligned horizontally, with the same results as in Figure 3. Therefore, it was concluded that for the investigated gyroscopic samples the X and Z axes are noisier than the Y axis [11]. The examinations results show that MEMS sensors of the IMU "InvenSense MPU-6050 type" are not highly accurate. So, this module can be used as the primary means for determining movement and orientation parameters when high precision conditions are not posed.

## 5. Calibration of the Gyroscope InvenSense MPU-6050 

**Figure 4:**
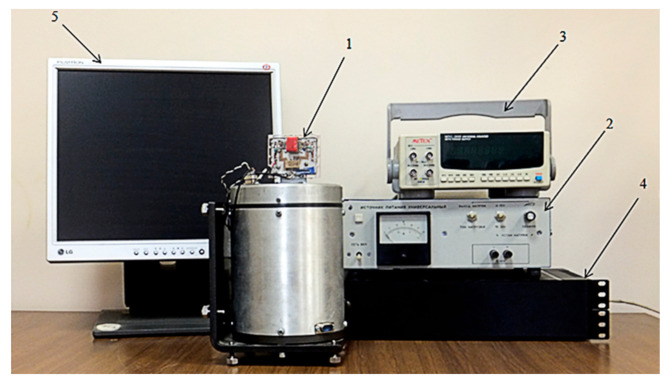
Experimental equipment: 1 is the mini-centrifuge with the exemplary rotary device and the IMU MPU-6050 breadboard; 2 is the PSM-6003 power source; 3 is the frequency meter METEX MXC-260; 4 is the electronic block; and 5 is the computer.

A calibration procedure is required to improve the accuracy of determining the motion and orientation parameters with inertial sensors. Experimental equipment used for calibration consists of a uniaxial mini-centrifuge with an exemplary rotary device and the IMU “MPU-6050” 1 breadboard, connected to a power source “PSM-6003” 2 (accuracy (programming) is 0.05% + 5 mV offset, stability is 0.02% + 1 mV offset, temperature coefficient per °C is 0.01% + 3 mV offset), which sets supply voltage in the range 2.4–3.4 V, a frequency meter “METEX MXC-260” 3 (short-term stability is ±3 × 10^−9^/s, long-term stability is ±2 × 10^−5^/month, temperature coefficient is ±5 × 10^−6^ in a range of (0–+40) °C), an electronic unit 4 and a computer 5 (Figure 4). The step of rotation angle change is 10° in the range from 0° to 360° (error of angle setting is 0.08%), and the mini-centrifuge allows setting different values of the angular velocity in the range from 0 to 240°/min. in both rotation directions with relative error up to 0.1%.

An ideal output signal from the gyroscope should be proportional to the projection of the absolute rotation angular velocity *ω* on the corresponding axis (for example $Z-U_{\omega z}$):

(14) $$U_{\omega z}=k_{z}\omega_{z},$$

where $k_{z}$ is the total transmission coefficient (scale factor), which depends on the gyroscope transmission ratio, as well as on the transmission ratio of the amplifier and other transducers of the measuring circuit.

In fact, each gyroscope has its own scale factor, bias instability, cross-links and other parameters, so we will use the following model of a gyroscope output signal:

(15) $$U_{\omega z}=k_{z}\omega_{z}+k_{zx}\omega_{x}+k_{zy}\omega_{y}+U_{\omega z0}+\xi_{\omega z},$$

where $k_{zx},\;k_{zy}$ are the cross-sensitivity coefficients; $U_{\omega z0}$ is the gyroscope zero offset; $\xi_{\omega z}$ is the measurement noise.

Angular velocity sensors can be constructed by different principles, so the output signal model (15) is specified with additional components. Thus, all electromechanical gyroscopes, including MEMS, are characterized by a significant sensitivity of the zero offset to a linear acceleration. Therefore, for them, the of the output signal model is specified by adding components proportional to the projections $a_{x},\;a_{y},\;a_{z}$ of apparent acceleration on the gyroscope sensitivity axis:

(16) $$U_{\omega z}=k_{z}\omega_{z}+k_{zx}\omega_{x}+k_{zy}\omega_{y}+U_{\omega z0}+b_{zx}a_{x}+b_{zy}a_{y}+b_{zz}a_{z}+\xi_{\omega z},$$

where $b_{zx},\;b_{zy},\;b_{zz}$ are coefficients of the gyroscope zero signal sensitivity to accelerations (so called "drift from g").

The task of calibrating the gyroscope means determining parameters of its output signal model (16) $k_{z},\;k_{zx},\;k_{zy},\;U_{\omega z0},\;b_{zx},\;b_{zy},\;b_{zz}$ on order to consider them when calculating the true value of the measured angular velocity.

To obtain data for calibration, the gyroscope is set on the optical dividing head (ODH) according to provisions given in [3], considering that the vector $\vec{g}$ is pointed vertically down. Data is read during a minute when the sensor rotates about an axis with 90° step. The orientation system outputs a 100 Hz signal, which is a one-dimensional array of 10 elements. The first three elements of the array are data from a triple axis accelerometer. During the reading process, the data is written to a text file as a spreadsheet to be later imported into mathematical packets and calculated. To reduce the effects of measurement noise $\xi_{\omega z}$ the output signal of the gyroscope is averaged over the measurement time (1 min), so it is not taken into account during calibration.

According to model (16), the gyroscope output signal depends on the angular velocity projections $\omega_{x},\;\omega_{y},\;\omega_{z}$ and accelerations $a_{x},\;a_{y},\;a_{z}$. That is why for the calibration the test values of angular velocity *ω* and acceleration *a* have to be set. Therefore, the gyroscope calibration is divided into two stages. On the first stage the angular rotation velocity is specified, on the second stage the fixed gyroscope is set in different positions relative to the gravity acceleration vector $\vec{g}$, as with a calibration of accelerometers. Thus, on the first stage coefficients $k_{z},\;k_{zx},\;k_{zy}$ are determined and on the second stage coefficients $U_{\omega z0},\;b_{zx},\;b_{zy},\;b_{zz}$ are determined.

On the first calibration stage, the angular velocities of gyroscope rotation are set without changing its position relative to the gravity acceleration vector $\vec{g}$. Therefore, the gyroscope output signal model at this stage looks like

(17) $$U_{\omega z}=k_{z}\omega_{z}+k_{zx}\omega_{x}+k_{zy}\omega_{y}+U_{\omega z0\Sigma },$$

where $U_{\omega z0\Sigma }=U_{\omega z0}+b_{zx}a_{x}+b_{zy}a_{y}+b_{zz}a_{z}$ is the total gyroscope zero offset.

When the gyroscope is calibrated only through a direct measurement channel (relative to the *Z* axis), the model of its output signal is $U_{\omega z}=k_{z}\omega_{z}+U_{\omega z0\Sigma }$. To calculate parameters $k_{z}$ and $U_{\omega z0\Sigma }$, two angular velocity values $\omega_{z}$ are specified and two linear equations are obtained, their solving yields the following results:

(18) $$k_{z}=\frac{\sum_{i=1}^{N}\omega_{zi}\cdot \sum_{i=1}^{N}U_{\omega zi}-N\cdot \sum_{i=1}^{N}\left(\omega_{zi}U_{\omega zi}\right)}{{\left(\sum_{i=1}^{N}\omega_{zi}\right)}^{2}-N\cdot \sum_{i=1}^{N}\omega_{zi}^{2}};\;U_{\omega z0\Sigma }=\frac{1}{N}\left(\sum_{i=1}^{N}U_{\omega zi}-k_{z}\cdot \sum_{i=1}^{N}\omega_{zi}\right)\;.$$

According to the results of calculations in MatLab, on the first stage the following values of the coefficients received are: $k_{x}=-14.532824;$ $k_{y}=-14.352421;$ $k_{z}=-14.342493;$ $U_{\omega x0\Sigma }=-373.611859;$ $U_{\omega y0\Sigma }=300.418374;$ and $U_{\omega z0\Sigma }=-166.451145.$

The gyroscope zero signal sensitivity to acceleration (“drift from *g*”) is

(19) $$b_{zy}=\frac{U_{\omega z4}-U_{\omega z2}}{2g};\;b_{zz}=\frac{U_{\omega z1}-U_{\omega z3}}{2g};\;U_{\omega z0}=\frac{1}{4}\sum_{i=1}^{4}U_{\omega zi}.$$

According to the results of calculations in MatLab, on the second stage the following values of the coefficients received are: $b_{xz}=-0.021594;$ $b_{xx}=0.005698;$ $U_{\omega x0}=-260.293125;$ $b_{yx}=0.049215;$ $b_{yy}=0.059203;$ $U_{\omega y0}=225.423762;$ $b_{zy}=0.025289;$ $b_{zz}=0.057872;$ and $U_{\omega z0}=-116.683324.$

Let us calculate calibrated values of the gyroscope output signal $\omega_{G}$ and determine measurement errors of the gyroscope angular velocity $\Delta \omega_{i}$. For simplicity, we assume that the rotation of the gyroscope is given only around one axis Z ($a_{x}=a_{y}=0;\;a_{z}=g$). At the same time for each value $\omega_{zi}$ of the rotary stand we determine the calibrated values of the gyroscope output signal:

(20) $$\omega_{G}=\omega_{z}=\frac{U_{\omega z}-U_{\omega z0}-b_{zz}g}{k_{z}}.$$

The absolute measurement error for the angular velocity of the gyroscope during calibration is determined by the ratio $\Delta \omega_{i}=\omega_{zi}-\omega_{Gi}$. Results of the calculations are presented in Table 2.

## 6. Analysis of Output Signals of InvenSense MPU-6050

**Figure 5:**
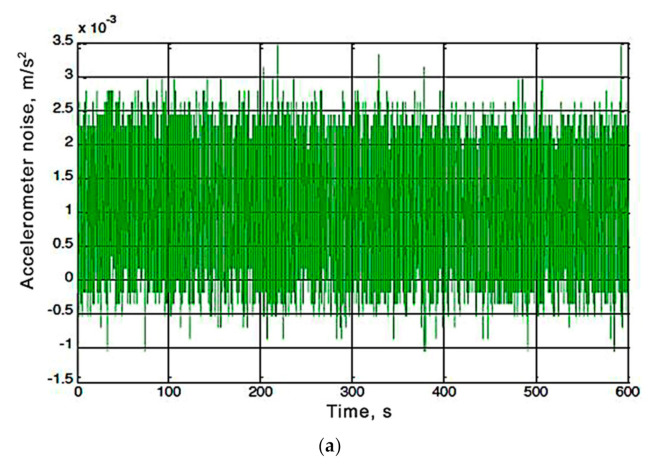

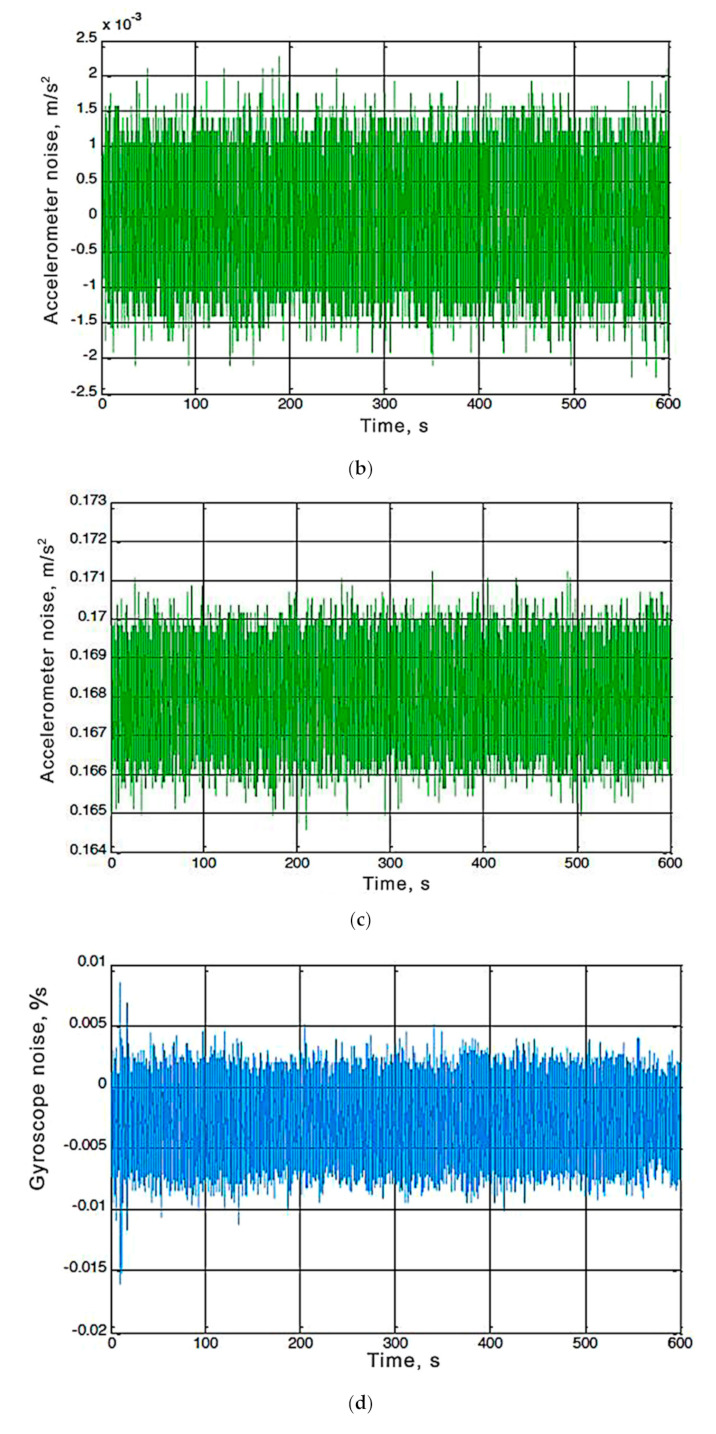

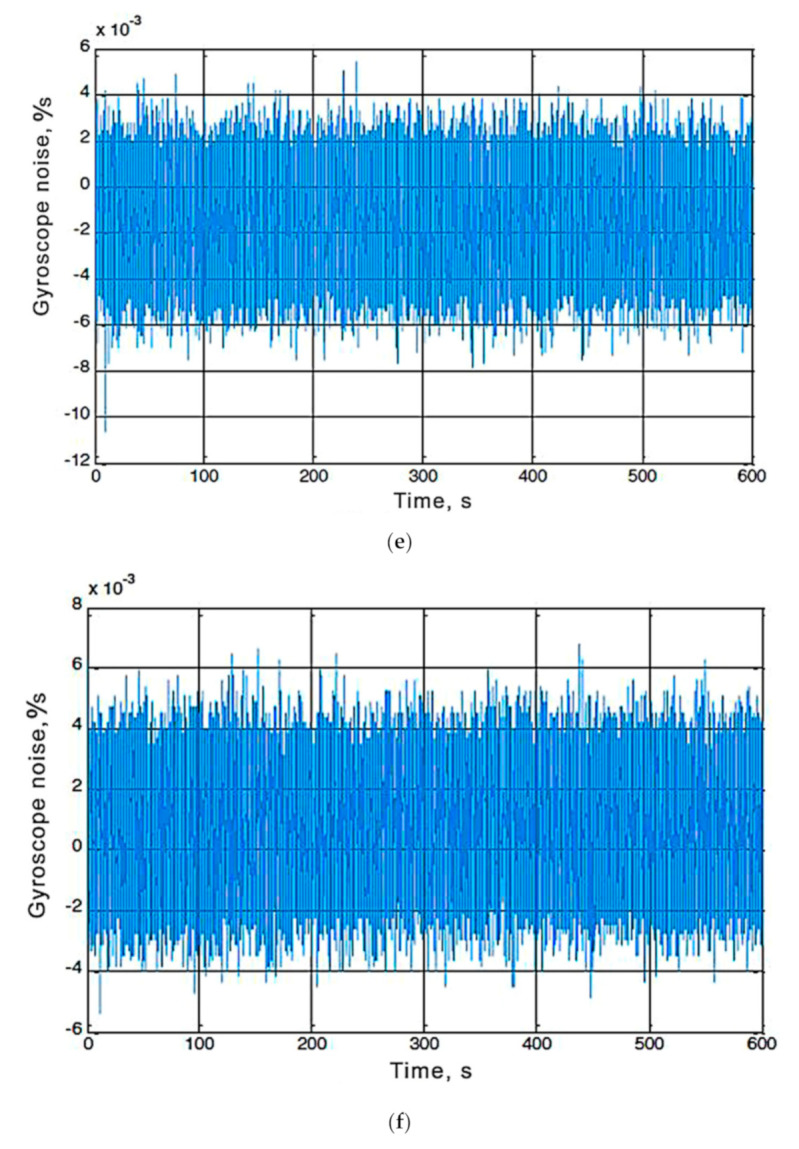
Noise of the output signals from the accelerometer ((**a**) is X-axis, (**b**) is Y-axis, and (**c**) is Z-axis) and from the gyroscope ((**d**) is X-axis, (**e**) is Y-axis, and (**f**) is Z-axis) of the MPU-6050 sensor.

To estimate noise of accelerometer and gyroscope output signals, the MatLab application was used. The calibrated values of the sensor were being read during 10 min, after this time all the results were imported into a one-dimensional array. To calculate the standard deviation of the array elements in MathLab a default function *std()* was used [28,29]. The noisy output signals of the calibrated accelerometer and gyroscope for each axis are shown in Figure 5.

Analyzing the dependencies (Figure 5), we conclude that the output signals for most axes of the sensor are offset relative to zero. Such offset should be taken into account in following calculations by introducing constant correction coefficients.

**Figure 6:**
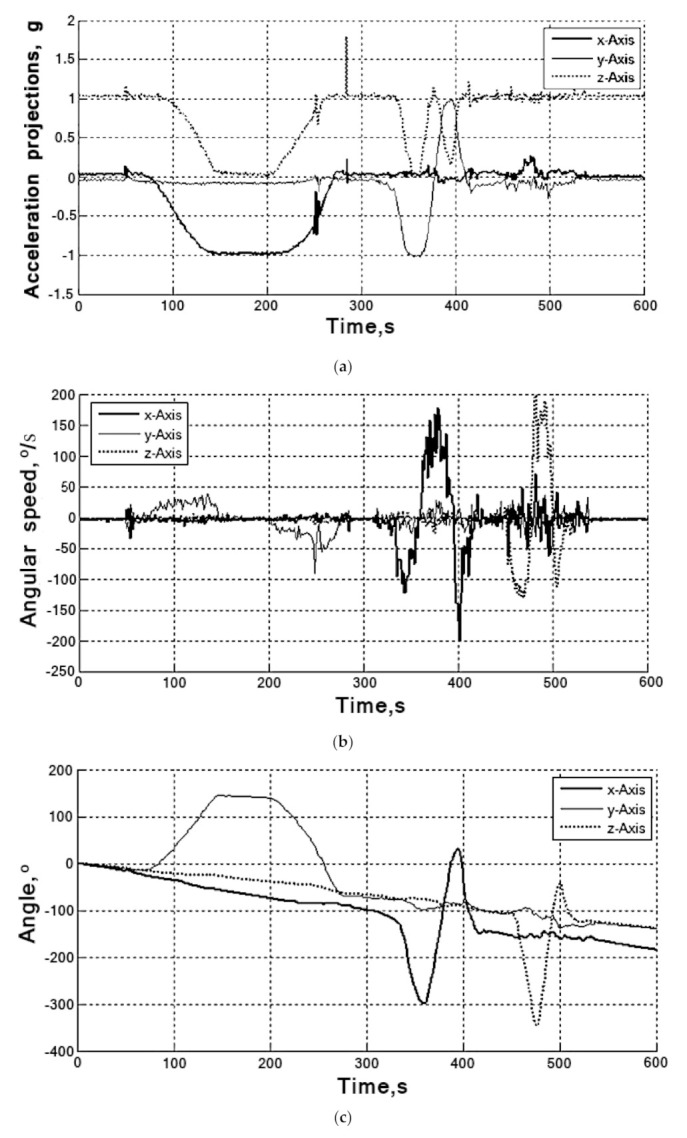
MPU-6050 sensor readings on axes of the accelerometer (**a**), on axes of the gyroscope (**b**), and data about angle rotations while integrating the gyroscope readings (**c**).

Data were read from registers of the MPU-6050 sensor at a frequency 10 Hz [30]. The gyroscope readings are integrated by a microcontroller to determine the angular velocities relative to the three axes (results are shown in Figure 6). In Figure 5c shows how the error is being accumulated while integrating readings. This is so-called zero drift, with the sensor being fixed and the angle value increasing at almost constant speed). In Figure 6a it can be seen that the accelerometer readings are noisy with high frequency interference, which leads to errors in calculation of deflection angles.

Analysis of the dependence (Figure 5) shows that the output signals of most sensor’s axes are significantly offset from zero and noisy by high frequency interference. Such offset should be taken into account in calculations by introducing constant correction coefficients. When the angular velocity is integrated, the angle is determined wrongly (Figure 6) due to the error accumulation (low-frequency noise) and accelerometer’s making a high-frequency interference. Therefore, to reduce the effects of noise, the sensor data (accelerometer and gyroscope) must be processed with an alpha-beta filter or the Kalman filter [31,32,33].

## 7. Conclusions

We analyzed in this paper the gyroscope random error components such as bias instability and random rate walk, as well as those cause by the presence of white and exponentially correlated (Markov) noise. The MEMS gyroscopes of InvenSense MPU-6050 type were investigated at each axis with a sampling frequency 70 Hz, as a result, the Allan deviation curves were plotted. The Allan deviation curves allowed determining the values of the instability coefficient for zero (the minimum value for the *Y*-axis is B_Y_ = 0.001277°/s; the maximum value for the *Z*-axis is B_Z_ = 0.002449°/s) and angle random walk (the minimum value for the *X*-axis is θ_ARW.X_ = 0.009145°/s/√Hz; the maximum value for the *Y*-axis is θ_ARW.Y_ = 0.009997°/s/√Hz) for each axis, and finding out that the output signals of the gyroscopes have no Markov noise (there is no local maximum between asymptotes with inclinations +0.5 and −0.5 in Figure 3) and random rate walks (there are no asymptotes with inclinations +0.5 in Figure 3, because increase of the averaging time was not analyzed). Similar studies were performed, the accuracy of determining the scale factors of gyroscopes was checked, and X and Z axes were concluded to be are noisier than Y axis.

During the process of IMU calibration, the correction coefficients were calculated. At the first stage of calibration, a scale factor of the gyroscope and coefficients of cross sensitivity were determined at given values of the rotation angular velocity. At the second stage of calibration, the gyroscope zero was offset and the sensitivity coefficients of the gyroscope zero signal to acceleration sensitivity were determined for different positions of the gyroscope relative to the gravitational acceleration vector. Correction coefficients provide partial compensation of the influence of destabilizing factors. Moreover, they provide determination of inaccuracy of the perpendicularity of the sensitivity axes, the conversion coefficients for each axis, transform the sensor output codes into units of measured value and also the zero offset for each axis. The output signals of the calibrated gyroscope were read in 10 min and imported into MatLab to calculate the standard deviation of the array elements. These signals are noisy and there is an offset from zero on all axes. Z-axes of the gyroscope and accelerometer have the maximum values of zero offset and standard deviation of noise, while y-axes have the minimum ones. Data of the accelerometer and gyroscope should be processed by the alpha-beta or Kalman filter to reduce noise.

The determined parameters of the output signal model of the IMU gyroscope may be employed for integrating into a program code based on modern microcontrollers of the AVR family. This will increase accuracy of algorithms based on the Kalman or Madgwick digital filters with relatively high sampling frequencies about 100 Hz. Further studies will focus on determining the optimal averaging time when calculating the Allan variance considering the processing of inertial sensor information at long runs. This is important for calibrating inertial sensors and will increase efficiency of noise structure identification in channels of measuring equipment.

## Figures and Tables

**Table 1:** Electrical characteristics of the InvenSense MPU-6050.

Parameter	Conditions	Min	Typ	Max	Units
**Gyroscope Sensitivity**					
Full-Scale Range	FS_SEL = 0 (3)	±250		±2000	°/s
Gyroscope ADC Word Length			16		bits
Sensitivity Scale Factor	FS_SEL = 0 (3)	16.4		131	LSB/(°/s)
Sensitivity Scale Factor Tolerance	25 °C	−3		+3	%
Sensitivity Scale Factor Variation Over Temperature			±2		%
Nonlinearity	Best fit straight line; 25 °C		0.2		%
Cross-Axis Sensitivity			±2		%
**Gyroscope Zero-Rate Output (ZRO)**					
Initial ZRO Tolerance	25 °C		±20		°/s
ZRO Variation Over Temperature	−40 °C to +85 °C		±20		°/s
Power-Supply Sensitivity	100 mVpp; VDD = 2.5 V	0.2		4	°/s
Linear Acceleration Sensitivity	Static		0.1		°/s/g
**Gyroscope Noise Performance**	FS_SEL = 0				
Total RMS Noise	DLPFSFG = 2 (100 Hz)		0.05		°/s-rms
Low-frequency RMS Noise	Bandwidth 1 Hz to 10 Hz		0.033		°/s-rms
Rate Noise Spectral Density	At 10 Hz		0.005		°/s/√Hz
**Gyroscope Start-Up Time**	DLPFCFG = 0				
ZRO Setting (from power-on)	to ±1°/s of Final		30		ms
**Accelerometer Sensitivity**					
Full-Scale Range	AFS_SEL = 0 (3)	±2		±16	g
Accelerometer ADC Word Length	In two’s component format		16		bits
Sensitivity Scale Factor	AFS_SEL = 0 (3)	2.048		16.384	LSB/g
Initial Calibration Tolerance			±3		%
Sensitivity Change vs. Temperature	−40 °C to +85 °C		±0.02		%/°C
Nonlinearity	Best fit straight line; 25 °C		0.5		%
Cross-Axis Sensitivity			±2		%
**Zero-G Output**					
Initial Calibration Tolerance	X and Y axes		±50		mg
	Z axis		±80		mg
Zero-G Level Change vs. Temperature	X and Y axes, 0 °C to +70 °C Z axis, 0 °C to +70 °C		±35 ±60		mg mg
**Accelerometer Noise Performance**					
Power Spectral Density	@10 Hz, ODR = 1 kHz		400		μg/√Hz
Intelligence Function Increment			32		mg/LSB

**Table 2:** Determination of accuracy of gyroscope calibration on axes.

Angular Velocity Value *ω_zi_*, ^o^/s	Measured Calibration Value of Angular Velocity *ω_Gi_*, ^o^/s			Absolute Measurement Error for the Gyroscope Angular Velocity during Calibration Δ*ω_i_*, ^o^/s		
	Axis X	Axis Y	Axis Z	Axis X	Axis Y	Axis Z
−150	−149.7543	−149.9781	−149.8478	−0.2457	−0.0219	−0.1522
−120	−119.6212	−119.6256	−119.6116	−0.3788	−0.3744	−0.3884
−100	−100.2933	−100.3465	−100.2131	0.2933	0.3465	0.2131
−80	−79.9559	−80.0618	−80.0617	−0.0441	0.0618	0.0617
−60	−60.0673	−59.9267	−60.1414	0.0673	−0.0133	0.1414
−40	−39.8056	−39.8653	−39.9171	−0.1944	−0.2347	−0.0829
−20	−19.6373	−19.7184	−19.8632	−0.3427	−0.2816	−0.1368
0	0.013588	−0.003654	0.007436	−0.013588	0.003654	−0.007436
20	20.2334	20.0129	19.8463	−0.2334	−0.0129	0.1537
40	39.9792	39.9491	39.9158	0.0208	0.0509	0.0842
60	60.1866	60.1149	60.2033	−0.1866	−0.1149	−0.2033
80	80.1018	80.0238	80.0788	−0.1018	−0.0238	−0.0788
100	100.5863	100.3816	100.3497	−0.5863	−0.3816	−0.3497
120	119.9062	119.7367	119.9022	0.0938	0.2633	0.0978
150	150.0168	150.0816	150.0365	−0.0168	−0.0816	−0.0365
